# A Cross-Sectional Study of the Current Management of Hypertriglyceridemia

**DOI:** 10.7759/cureus.20732

**Published:** 2021-12-27

**Authors:** Raghad A Jar, Ealaf Melibari, Nidaa Almehmadi, Renad O Kalantan, Mohamed E Ahmed, Abdulhalim J Kinsara

**Affiliations:** 1 Medicine and Surgery, King Saud Bin Abdulaziz University for Health Sciences College of Medicine, Jeddah, SAU; 2 Biostatistics, King Abdullah International Medical Research Center, Jeddah, SAU; 3 Cardiology, King Saud Bin Abdulaziz University for Health Sciences, Jeddah, SAU

**Keywords:** saudi arabia, management, outcome, coronary heart disease, dyslipidemia, triglyceride

## Abstract

Objectives

To estimate the prevalence of hypertriglyceridemia (HTG), determine the association between HTG and the risk of ischemic heart disease and major adverse cardiovascular events. Lastly, to assess the management outcomes of HTG in terms of the different drugs in the treatment plan.

Methods

A retrospective, longitudinal study at a tertiary hospital was conducted. All who came in were screened. Patients with HTG (TAG [triacylglyceride] 2.3 mmol/L) in the last five years were included in the study. The data included the demographic variables, potential causes, and the methods of management. All data were recorded in a standard data collection form and analyzed by an appropriate statistical tool, using the John Macintosh Project (JMP) software version 15 (Cary, NC: SAS Institute Inc.).

Results

Of 300 patients included, 174 (58.0%) were male, with a mean age of 57.8±13.4 years. Pre-treatment, the mean triglycerides (TG) was 3.2±2.3 mmol/L, low-density lipoprotein (LDL) 2.7±1.3 mmol/L, high-density lipoprotein (HDL) 0.93±0.30 mmol/L, and the total cholesterol (TC)was 5.2±1.3 mmol/L. All the patients have prescribed a statin, 144 (48.0%) received aspirin, six (2.0%) fenofibrate, and three (1.0%) gemfibrozil. At the follow-up, the level of the TG was 2.6±1.3 mmol/L (P=0.001), LDL 2.5±1.2 mmol/L (P=0.006) and total cholesterol (TC) 4.7±1.5 mmol/L (P=0.001). Almost a third (28.2%) developed cardiac complications, five (1.6%) presented with unstable angina, six (2.0%) as non-ST segment elevation myocardial infarction (NSTEMI), three (1.0%) had ST segment elevation myocardial infarction (STEMI), and 19 (6.3%) had heart failure. A small proportion (17.3%) had a percutaneous coronary intervention, 27 (9.0%) had single-vessel disease, 12 (4.0%) two-vessel disease, and 13 (4.3%) three-vessel disease.

Conclusions

Many physicians do not pay attention to HTG in everyday practice, although HTG contributes significantly to the occurrence of coronary heart disease. In our study, the majority had mixed hyperlipidemia. One-third of patients with high triglycerides developed ischemic heart disease. The use of fenofibrate and gemfibrozil was not high. A low occurrence of pancreatitis was noted in our series.

## Introduction

Cardiovascular diseases and atherosclerosis have been associated with low-density lipoprotein cholesterol (LDL-C) levels in the last decade, but growing evidence supports the positive relationship between cardiovascular diseases and high levels of triglycerides (TG) [1]. Severely elevated TG is associated with pancreatitis but other systems may also be affected. TG elevation could be primary or familial or due to a secondary cause, such as uncontrolled diabetes, obesity, or alcohol consumption. A high TG, in many cases, is asymptomatic until it exceeds 500 - 1,000 mg/dL (5.7 mmol/L or above) [2]. Recently, TG was considered a major independent risk related to LDL-C or to its lowering therapy [3]. The prevalence of high TG in Saudi Arabia is estimated at 40.3% [4].

It is debatable whether the reduction of TG will prevent cardiovascular risks [5-6]. Our study focused on the association of TG as an independent risk factor for cardiovascular disease (CVD) in Saudi adults. The study also determined the effect of the current management in lowering the level of high TG.

## Materials and methods

This was a retrospective, longitudinal study conducted at a tertiary care center in Saudi Arabia, in the period 2015-2020. The participants were selected consecutively from the hospital’s electronic medical record system. All patients aged > 18 years included. Those with incomplete data (42 patients ) were excluded. The study incorporated all the patients with a TG > 2.3 mmol/L over the last five years. The demographic characteristics, including the comorbidities, medication, and complications related to a high TG profile were collected. In total, 300 patients were enrolled in the study. All the desired information was recorded in a standard Excel sheet (Redmond, USA: Microsoft Corporation). Acute pancreatitis was defined by (a) abdominal pain suggestive of pancreatitis, (b) serum amylase or lipase level greater than three times the upper normal value, or (c) characteristic imaging findings of acute pancreatitis. Acute kidney injury was defined by measuring the abrupt (≤48 hours) reduction of kidney function: increased serum creatinine levels 1.5-fold from baseline. Cardiac disease was recorded if the patient had chest pain with ECG changes and rise in troponin, and undergone cardiac catheterization. Death due to a cardiac cause was included as a measured outcome.

This study was approved by the Institutional Review Board of King Abdullah International Medical Research Center. Frequency and percentage were used to describe the categorical variables. The Chi-square or Fisher’s exact test was used to compare the categorical data. A p-value of less than 0.05 was considered statistically significant. The data were analyzed using the John Macintosh Project (JMP) software version 15 (Cary, NC: SAS Institute Inc.).

## Results

A total of 342 patients were identified as TG >2.3 mmol/l. The sample realized as 300, with a mean age of 57.8±13.4 years. 42 patients were excluded due to incomplete data. More than half (58.0%, n=174) were male. Of the female group, six (2.0%) were pregnant. The mean body mass index (BMI) was 31.1±6.5 kg/m2. A small proportion (4.7%, n=14) had a family history of high TG, 31 (10.3%) were smokers, and 17 (5.7%) were ex-smokers. Only one (0.3%) reported alcohol intake.

The pre-treatment TG at the first visit before taking any medication were TG 3.2±2.3 mmol/L, with the low-density lipoprotein (LDL) 2.7 ±1.3 mmol/L, high-density lipoprotein (HDL) 0.93 ±0.30 mmol/L, and the total cholesterol (TC) was 5.2 ±1.3 mmol/L. The mean liver profiles were alanine aminotransferase (ALT) 27.3±20.8 U/L, aspartate aminotransferase (AST) 21.1±10.2 U/L, gamma-glutamyltransferase (GGT) 52.1±72.6 U/L and bilirubin 9.2±5.8 μmol/L (Table 1). More than a third, 112 (37.3%), were on a calcium channel blocker, 93 (31.0%) on beta-blockers, 80 (26.7%) on angiotensin-converting enzyme (ACE) inhibitors, 80 (26.7%) on angiotensin receptor blockers (ARB), 37 (12.3%) and only one (0.3%) were on tamoxifen. A small proportion (8.7%, n=26) was taking cortisone and 16 (5.3%) were on antipsychotic medication (Table 2). 

**Table 1:** Lipid profile and liver enzymes before and after treatment TG: Triglycerides; LDL: Low-Density Lipoprotein; HDL: High-Density Lipoprotein; TC: Total Cholesterol; ALT: Alanine Transaminase; AST: Aspartate Aminotransferase; GGT: Gamma-glutamyl Transferase.

Parameter	Pre-treatment	Post-treatment	P value
TG mmol/L	3.2 ±2.3	2.6 ±1.3	0.001
LDL mmol/L	2.7 ±1.3	2.5 ±1.2	0.006
HDL mmol/L	0.93 ±0.30	0.98 ±0.38	0.009
TC mmol/L	5.2 ±1.3	4.7 ±1.5	0.001
ALT U/L	27.3 ±20.8	24.9 ±22.7	0.039
AST U/L	21.1 ±10.2	19.9 ±11.9	0.197
GGT U/L	52.1 ±72.6	45.9 ±60.4	0.326
Bilirubin μmol/L	9.2 ±5.8	11.5 ±28.8	0.219

**Table 2:** Participant treatment ACEI: Angiotensin-converting enzyme Inhibitors; ARBs: Angiotensin Receptor Blockers; CCB: Calcium Channel Blocker; HIV prot: HIV Protease Inhibitors.

Medication	N (%)
Nitrates	37(12.3)
Beta blockers	93(31.0)
ACEI	80(26.7)
ARBs	80(26.7)
Aspirin	144(48.0)
CCB	112(37.3)
Cortisone	26(8.7)
Antipsychotic	16(5.3)
Estrogen	0(0)
Retinoid	0(0)
HIV Prot	3(1.0)
Thiazide	3(1.0)
Tamoxifen	1(0.3)
Fibrates	9 (3.0)
Gemfibrozil	0(0)
Statin	219(73.0)
Niacin	0(0)

At follow-up, there was a significant decrease in the TG, LDL, and TC with the management. The mean TG was 2.6±1.3 mmol/L (P=0.001), LDL 2.5±1.2 mmol/L (P=0.006), and the TC was 4.7±1.5 mmol/L (P=0.001), HDL 0.98±0.38 mmol/L (P=0.009), indicating a significant increase in the HDL. The different medications used did not adversely affect the liver profiles. The post-treatment AST was 19.9±11.9 U/L (P=0.197), GGT 45.9±60.4 U/L (P=0.326), and the bilirubin was 11.5±28.8 μmol/L (P=0.219), while the ALT decreased to 24.9±22.7 U/L (P=0.039).

A small proportion (2.3%, n=7) had familial dyslipidemia and one (0.3%) had clinical xanthoma. Of the sample, the most frequently found systemic disease was type 2 diabetes mellitus (DM) (72.0%, n=216), and five (1.4%) had type 1 DM. Hypothyroidism occurred in 68 (22.7%), kidney disease/hemodialysis in 74 (24.7%), autoimmune diseases in 16 (5.3%), and thyrotoxicosis in three (1.0%) (Table 3). Cardiac disease was noted in 28.2%. Of the group who underwent a percutaneous coronary intervention, 27 (9.0%) had single-vessel disease, 12 (4.0%) two-vessel disease, 13 (4.3%) three-vessel disease (Table 4).

**Table 3:** Comorbidities in the sample AKI: Acute Kidney Injury; CKD: Chronic Kidney Disease; SLE: Systemic Lupus Erythematous; RA: Rheumatoid Arthritis.

Outcome	N (%)
Pancreatitis	
Yes	3(1.0)
Renal function	
AKI	5(1.7)
CKD	54(18.0)
Hemodialysis	15(5.0)
Proteinuria	
Yes	47(15.7)
Thyroid Disease	
Thyrotoxicosis	3(1.0)
Hypothyroidism	68(22.7)
Autoimmune	
SLE	1(0.3)
RA	4(1.3)
Other	11(3.7)
Long-term follow-up	
Lost follow-up	20(6.7)
Alive	266(88.6)
Dead	14(4.7)

**Table 4:** Complications occurring in the sample NSTEMI: Non-ST Elevation Myocardial Infarction; STEMI: ST Elevation Myocardial Infarction; CA 1VD: percutaneous coronary intervention of 1 vessel disease; CA 2VD: percutaneous coronary intervention of 2 vessel disease; CA 3VD: percutaneous coronary intervention of 3 vessel disease; DM 1: type 1 diabetes mellitus; DM 2: type 2 diabetes mellitus.

Variable	N (%)
Family dyslipidemia	
Yes	7(2.3)
Clinical Xanthoma	
Yes	1(0.3)
Atherosclerotic	
Ischemic stroke	17(5.7)
Hemorrhagic stroke	12(4.0)
Peripheral Artery Disease	00
Cardiac Disease	
Unstable angina	5(1.6)
NSTEMI	6(2.0)
STEMI	3(1.0)
CA 1VD	27(9.0)
CA 2VD	12(4.0)
CA 3VD	13(4.3)
Heart failure	
Yes	19(6.3)
DM	
DM 1	5(1.4)
DM 2	216(72.0)

The sample presented with various symptoms, including unstable angina five (1.6%), NSTEMI six (2.0%), STEMI three (1.0%), and 19 (6.3%) had heart failure. A small proportion (5.7%, n=17) had an ischemic stroke and 12 (4.0%) a hemorrhagic stroke. Only three (1.0%) of the participants had pancreatitis. At a mean follow-up of five years, the death rate was 14 (4.7%).

## Discussion

The association between cardiovascular diseases and atherosclerosis with the blood triglyceride level has been controversial as a risk factor [7,8,9,10]. Elevated TG association with an increased risk of atherosclerotic cardiovascular disease (ASCVD) becomes insignificant after adjusting for non-HDL cholesterol (non-HDL-C), which represents the total concentration of all Apo B-containing lipoproteins [11]. The fibrate lowering of TG reduces the risk of cardiovascular events when measured per unit change of non-HDL-C [12,13]. A systematic review and meta-analysis of observational studies evaluating hypertriglyceridemia (HTG) and LDL cholesterol (LDL-C) levels (hazard ratio 0.8; P=0.025) [14]. However, most studies suggested that hypertriglyceridemia is associated with a substantially increased long-term total mortality and CVD risk. No such data are available in developing countries, including Saudi Arabia. 

In our study, the groups with a high TG and TC were similar in terms of gender at the mid-fifties age range. This is comparable with a Turkish report, with a lipid abnormality recorded in 78.7% of men and 80.4% of women, and the highest prevalence in the 46-65 years age group [15].

One-quarter of our population had cardiac disease, all confirmed with coronary angiography. A varying spectrum of the coronary lesions was noted. The prevalence was similar to another study conducted in Riyadh, Saudi Arabia that suggested 20% of their sample had coronary heart disease [16]. The occurrence of the acute coronary syndrome spectrum was low, less than 2%. Reviewing the literature, we only found one case report of acute STEMI complicated by severe HTG [17]. 

The current study revealed that most of the current lipid therapy achieved the target goal with a significant reduction in the TG, LDL, and TC. A systematic review revealed statin can lower TG level (MD =-0.43; 95% CI: -0.76 to -0.09; Z =2.51; P=0.01) [18]. This effect is more marked with a potent statin. This approach is recommended by the guidelines. Statin treatment is recommended as the first drug of choice to reduce CVD risk in high-risk individuals with HTG ( >2.3 mmol/L or >200 mg/dL) [19] The use of fenofibrate and gemfibrozil was low in the sample, probably due to achieving the target with statins. The use of these two drugs was given class IIb [19]. It is important to stress the role of diet, exercise, and community-based intervention to decrease cardiac risk profile in patients with high TG [20]

The study will stimulate additional research in our region and other developing countries, as some of the findings may be related to the sample size. 

## Conclusions

High triglycerides level is an overlooked factor in the association between lipid parameters and coronary artery disease, which should be managed when evaluating the risk profile of patients. Our study indicated that a high TG presents mostly as chronic heart disease, rarely as an acute coronary syndrome. The current standard lipid therapy is an effective mode of therapy with a low incidence of pancreatitis. In our study, the majority had mixed hyperlipidemia. The use of fenofibrate and gemfibrozil was not high in our series.
